# A Case of Lactobacillus casei Endocarditis Associated With Probiotic Intake in an Immunocompromised Patient

**DOI:** 10.7759/cureus.38049

**Published:** 2023-04-24

**Authors:** Ali Rahman, Sura Alqaisi, Jayant Nath

**Affiliations:** 1 Internal Medicine, Mather Hospital/Northwell Health, Port Jefferson, USA; 2 Internal Medicine, Memorial Hospital Pembroke, Pembroke Pines, USA; 3 Imaging Cardiology, Memorial Hospital Pembroke, Pembroke Pines, USA

**Keywords:** probiotics, valve vegetation, immunocompromised hosts, endocarditis, lactobacillus casei

## Abstract

Probiotics are microorganisms, typically bacteria, similar to beneficial microbiota found in the human gut, usually consumed as dietary supplements or fermented foods. Although probiotics are generally safe, several cases of bacteremia, sepsis, and endocarditis associated with probiotics have been reported. Here we report a rare case of *Lactobacillus casei* endocarditis in a 71-year-old female, immunocompromised due to chronic steroid intake, who presented with a productive cough and low-grade fever. Blood cultures grew *L. casei *resistant to vancomycin and meropenem. Transesophageal echocardiography showed mitral and aortic vegetations; valve replacement was done after successfully removing vegetations. She was treated with a six-week course of daptomycin and recovered.

## Introduction

*Lactobacillus *endocarditis, or infection of the heart’s inner lining (endocardium) by *Lactobacillus* bacteria, is a rare condition typically associated with other underlying risk factors. Despite the increasing popularity of probiotic supplements and foods containing *Lactobacillus* bacteria, there is limited evidence to suggest that probiotic intake directly leads to *Lactobacillus *endocarditis. *Lactobacillus* is a genus of bacteria commonly found in the human gut and used as a probiotic in many fermented food products and dietary supplements. While probiotics have been touted for their potential health benefits, including improving digestive health and boosting the immune system, limited evidence supports these claims. Some studies have probed the benefits of probiotics in treating and preventing diarrhea associated with *Clostridium difficile*, irritable bowel syndrome, ulcerative colitis, Crohn’s disease, and eczema with promising results. Although no randomized trials have shown the benefit of routine probiotics, some meta-analyses have suggested probiotics may be beneficial in preventing *C. difficile*-associated diarrhea [1-4]. Despite limited evidence, probiotics are generally helpful as adjunctive therapy in treating recurrent *C. difficile*-associated diarrhea [5,6]. *Lactobacillus *endocarditis is caused by the entry of bacteria into the bloodstream and subsequent attachment to the heart valve or inner lining of the heart. This can occur through various means, including dental procedures, surgical procedures, and intravenous drug use.

While it is possible that probiotic supplementation could lead to *Lactobacillus *endocarditis, this is a rare occurrence and is typically associated with other underlying risk factors. Boyle and his co-workers proposed a list of two major risk factors that are immune compromise, including malignancy or debilitating state, and premature infants, and six minor risk factors that are central venous catheter, impaired intestinal barriers as in severe diarrhea or intestinal inflammation, the use of jejunostomy, concomitant administration of antibiotics a probiotic is resistant to, highly pathogenic probiotics and people with cardiac valvular diseases, for which *Lactobacillus* spp. are known to cause endocarditis [7]. Patients with one of the major risk factors or at least more than one of the minor risk factors are at high risk of developing sepsis, and using probiotics warrants caution. Most probiotic products contain strains of *Lactobacillus* that are unlikely to cause disease. The strains of *Lactobacillus* found in these products often differ from those associated with *Lactobacillus *endocarditis. In general, *Lactobacillus *endocarditis is a rare condition that affects individuals with underlying heart conditions, such as a heart valve abnormality or artificial heart valve. These individuals have an increased risk of developing bacterial infections in the heart, including Lactobacillus endocarditis. Few cases of endocarditis associated with *Lactobacillus* have been reported [8,9]. Here, we report *Lactobacillus casei*-associated endocarditis in an immunocompromised patient with native valves. This case is interesting because the *L. casei* implicated has never been previously reported to cause endocarditis in native valves. Moreover, *L. casei *was isolated in culture, and the outcome after six weeks of treatment was good, unlike other cases of suspected *Lactobacillus *endocarditis in immunocompromised patients.

## Case presentation

A 71-year-old female presented to the emergency department (ED) complaining of left calf pain, productive cough, and low-grade fever. Her symptoms started a week before her admission and progressively worsened, prompting her to come to the ED. She denied other symptoms. However, she endorsed taking over-the-counter probiotics containing 300 billion colony-forming units of *Lactobacillus casei *per gram for several months for better gut health; she took about 2 grams daily. She had no history of smoking or intravenous drug use. Her past medical history was remarkable for rheumatoid arthritis and diastolic heart failure. She was taking 15 mg of prednisone every morning and an injection of golimumab for her rheumatoid arthritis for at least a year.

At the ED, an assessment of her vital signs showed a temperature of 37.6°C, blood pressure of 100/70 mmHg, and heart rate of 100 beats per minute. The physical examination was remarkable for basal bilateral crackles on chest auscultation, and a pan systolic murmur of mitral regurgitation was picked at the cardiac apex, radiating to the axilla. The rest of the physical examination was unremarkable. Laboratory results are given in Table 1.

**Table 1:** Laboratory results WBC, while blood cell; BUN, blood urea nitrogen; ESR, erythrocyte sedimentation rate; SARS-CoV-2, severe acute respiratory syndrome coronavirus 2; PCR, polymerase chain reaction

Labs	Values	Normal range
WBC (×10^9^/L)	15	4.5-11
Hemoglobin (g/dL)	11	12-16
Platelets (×10^9^/L)	200	130-400
Neutrophils (%)	85	40-60
Lymphocytes (%)	8.2	20-40
Monocytes (%)	5	1.7-9.3
Eosinophils (%)	1	0-5
Basophils (%)	0.8	0-3
Sodium (mmol/L)	140	137-145
Potassium (mmol/L)	4	3.5-5.2
Chloride (mmol/L)	99	98-107
Carbone dioxide (mmol/L)	22	22-30
BUN (mg/dL)	11	7-17
Creatinine (mg/dL)	0.9	0.52-1.04
ESR (mm/hr)	400	0-29
D-dimer (ng/mL)	100	<250
Ferritin (ng/mL)	110	12-50
Fibrinogen (mg/dL)	220	200-400
SARS-CoV-2 PCR	Negative	Negative

**Figure 1:**
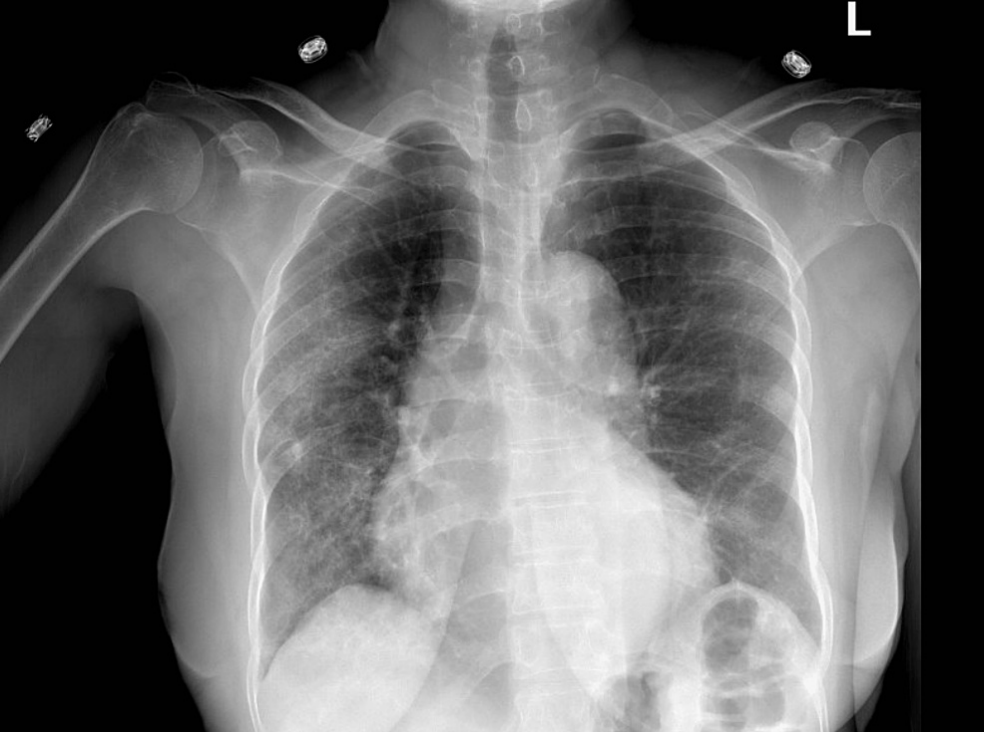
Chest X-ray showing cardiomegaly

**Figure 2:**
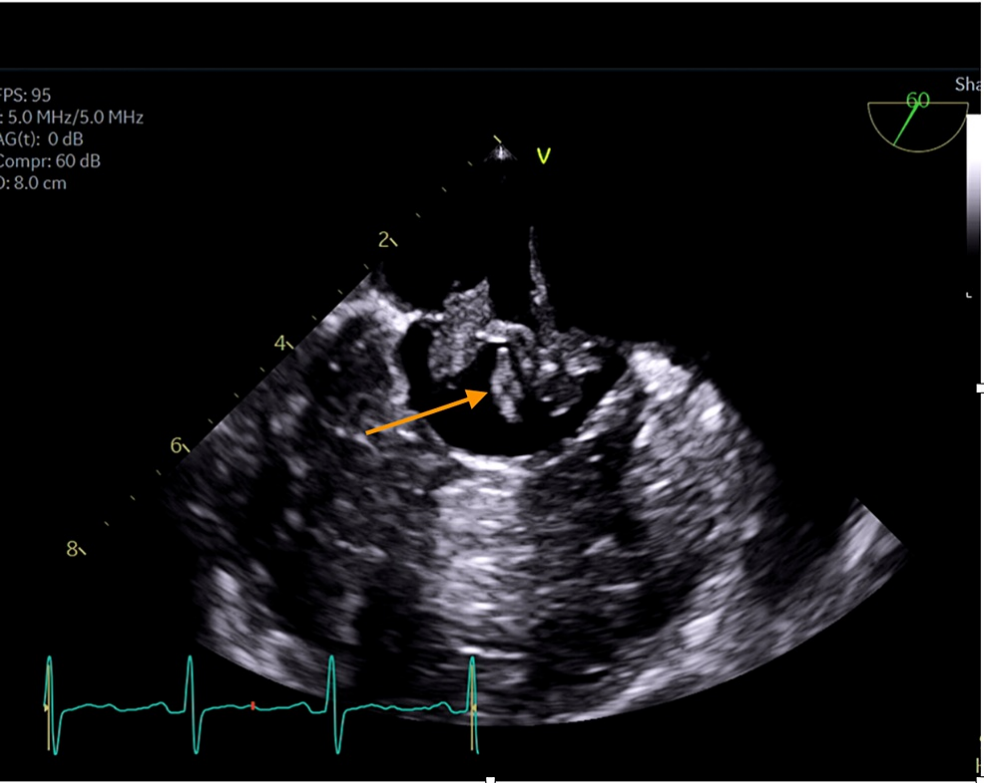
A TEE image showing multiple echodensities (arrow) on both sides of the mitral leaflet

**Figure 3:**
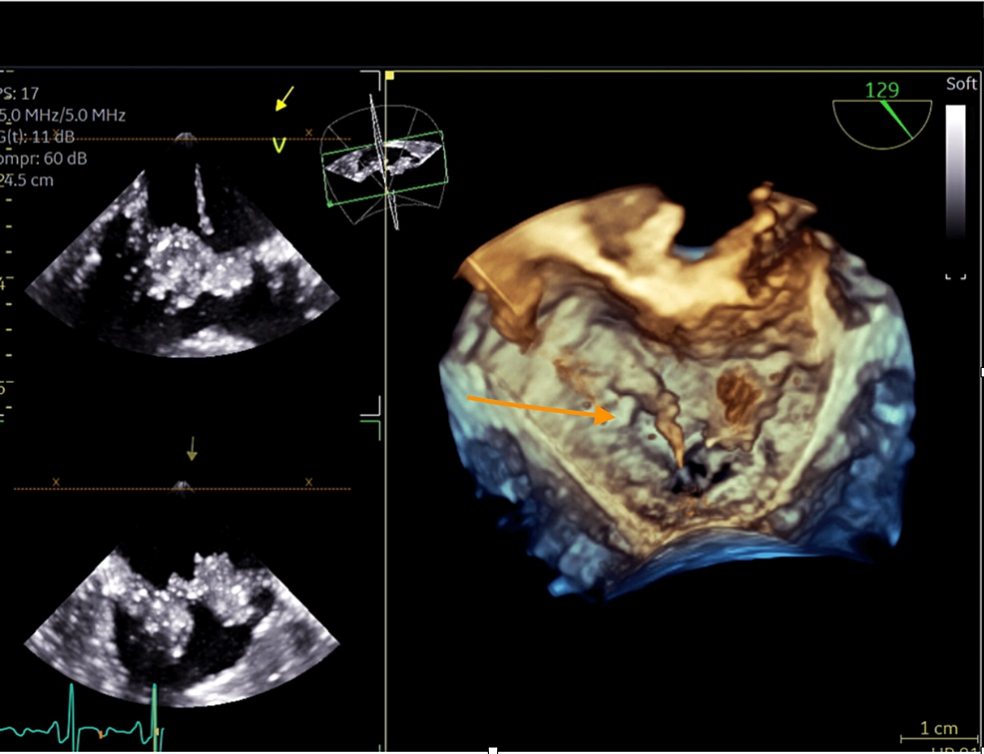
A three-dimensional image showing the mitral valve vegetation (arrow)

**Figure 4:**
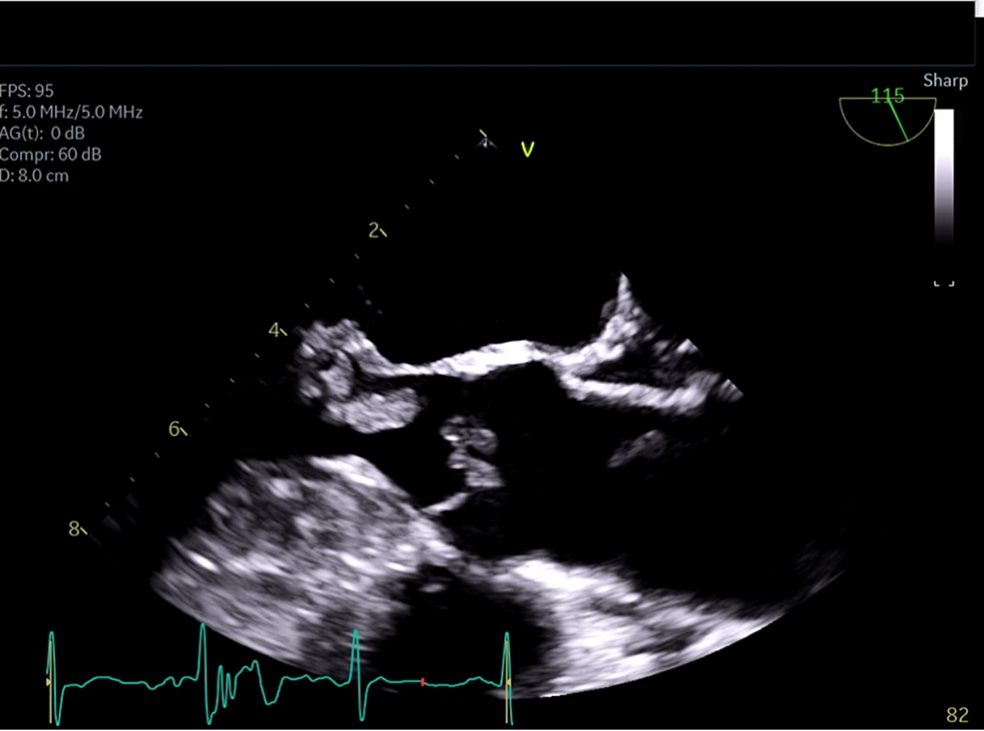
A TEE image showing the aortic valve vegetation

A chest X-ray revealed cardiomegaly (Figure 1). She had four blood culture samples taken, each taken from different sites. All yielded Gram-positive Coccobacilli (*L. casei*) resistant to meropenem and vancomycin, but susceptible to clindamycin, daptomycin, and linezolid as shown in the antimicrobial susceptibility test (Table 2). Doppler ultrasound of the left leg showed no features suggestive of deep venous thrombosis. The patient was evaluated by an infectious disease specialist who recommended transesophageal echocardiography (TEE). TEE showed multiple mobile echodensities on both sides of both mitral leaflets ranging from 1.0 to 1.5 cm, including one in the left ventricular outflow tract. There appeared to be a valvular abscess though no definite perivalvular abscess was seen. There was moderate mitral valve regurgitation but no mitral stenosis (Figures 2, 3). All three aortic leaflets were thickened, and echodensities were seen on the ventricular aspect of the aortic valve, suggestive of vegetation; no definitive periaortic abscess was seen and there was no aortic valve stenosis either (Figure 4).

**Table 2:** Antimicrobial susceptibility test

Antimicobial agent	Lactobacillus casei	Susceptibility
Clindamycin	0.25 ug/mL	Sensitive
Daptomycin	1.500 ug/mL	Sensitive
Linezolid	2.000 ug/mL	Sensitive
Meropenem	32.00 ug/mL	Resistant
Penicillin	0.750 ug/mL	Sensitive
Piperacillin/tazobactam	12 ug/mL	No interpretation
Vancomycin	256.000 ug/mL	Resistant

The patient's endocarditis was caused by *Lactobacillus casei*, likely from her probiotic intake and immunocompromised state. Empirical treatment with intravenous ampicillin 2 g every six hours for 14 days was initiated. Upon culture and sensitivity results, the patient was switched to intravenous daptomycin 560 mg once daily for six weeks. The cardiothoracic surgeon eventually reviewed the patient and recommended mitral and aortic valve replacements after successfully removing vegetation; the patient had aortic valve replacement with a 19-mm bioprosthetic valve and mitral valve replacement with a 25-mm bioprosthetic valve. There was no perivalvular abscess, and vegetation culture and histology did not yield* Lactobacillus casei*. She was given 5 mg warfarin daily to maintain her international normalised ratio in the range of 2.5-3.5. The patient was later discharged to a cardiac rehabilitation center.

## Discussion

The diagnosis of infective endocarditis is established in the presence of vegetation or intracardiac abscess demonstrating active endocarditis on histology or microorganisms confirmed by culture or histology of vegetation or intracardiac abscess. Modified Duke criteria are also widely used as a clinical criterion for diagnosing infective endocarditis. The role of TEE is based on its superior diagnostic value in terms of visualization and resolution, leading to higher sensitivity of approximately: 96% for native valves and 92% for prosthetic valves. TEE also has a sensitivity and specificity of close to 90% in detecting an endocardial abscess [10]. In this case, four blood culture samples were taken from different sites to isolate the organism and yielded *Lactobacillus casei *in all sets. Gram staining was done, which showed Gram-positive cocci in chains. *Lactobacillus* spp. is known to be Gram-positive, sometimes variable, as bacilli to coccobacilli, often occurring in chains; however, sometimes they may appear as short coccobacilli resembling Gram-positive cocci in chains.

Infective endocarditis is associated with many complications, which depend on the underlying commodities, the pathogen involved, and the course of an illness before diagnosis and treatment. Heart failure is the most common cardiac complication, mainly observed in *Staphylococcus aureus *infection. Despite systemic antibiotics' prescription, some patients continue to have a fever or ECG abnormalities, particularly those with a perivalvular abscess [11]. In our case, the fever subsided soon after the initiation of antibiotics. A perivalvular abscess increases the risk of septic embolization and a heart block, particularly when the aortic valve is involved [12]. Regarding the absence of ECG abnormalities in this case and the nature of the infecting organism, a perivalvular abscess was generally unlikely. A TEE was also confirmed, with a high sensitivity to detecting intracardiac abscesses [13].

Septic emboli occur more frequently with mitral vegetations than aortic, mainly when the vegetation is on the anterior mitral leaflet. The size of the vegetation and the microbiology were good predictors of the absence of septic emboli. A vegetation of more than 1.5 cm is known to predict one-year mortality. Likewise, unlike *Lactobacilli*, *S. aureus* infection and fungal endocarditis carry an increased risk of embolization [14]. In our case, vegetations were less than 1.5 cm, which was a good predictor of survival. Glomerulonephritis due to the deposition of immune complexes and interstitial nephritis due to commonly used drugs such as vancomycin are common renal complications. *Lactobacillus casei* isolated was resistant to vancomycin, and no renal complications were detected [15].

*Lactobacillus *endocarditis is a rare condition, and this case is notable for several reasons. First, the patient had no history of common risk factors for endocarditis. Second, *Lactobacillus* spp. has very low virulence, and reported bacteremia and associated sepsis are uncommon [16]. Moreover, the presence of *Lactobacillus* in blood cultures is particularly unusual. *Lactobacillus *is typically a commensal organism commonly found in the gut and not a widely known causative agent of endocarditis in native valves. However, the patient’s history of taking probiotics containing *Lactobacillus* for several months raises the possibility of the probiotics being a source of the infection, particularly concerning her immunocompromised state. Endocarditis is the most frequently reported infection associated with probiotics in adults, primarily with underlying risk factors. In children, cases of *Lactobacillus *bacteremia are observed, particularly in immunocompromised ones. Several case reports of *Lactobacillus *endocarditis have been reported in intravenous drug users, patients with prosthetic valves, valvular regurgitations, history of cardiac surgery, and immunocompromised patients [17]. The subject, in this case, was using prednisone and golimumab for her rheumatoid arthritis, which likely suppressed her immunity and increased vulnerability to *Lactobacillus* infection.

The clinical presentation of the patient was, however, less suspicious. Fever, mostly low-grade, is common in most cases of infective endocarditis. More fulminant symptoms of sepsis, congestive heart failure, or thromboembolic events are observed in acute cases. However, even subacute infective endocarditis, petechiae, splinter hemorrhage, Osler nodes, Janeway lesions, and Roth spots can be seen. Our subject did not show these signs, so the clinical suspicion of infective endocarditis was unlikely. The diagnostic challenge in this case also lies in distinguishing between a contaminant and a causative organism. The presence of *Lactobacillus *in the blood cultures can be a potential contaminant as it is a commonly occurring organism. However, the combination of the patient’s symptoms, physical exam findings, and imaging studies strongly suggests that *Lactobacillus *was the causative agent of endocarditis.

The diagnosis of *L. casei *bacteremia is difficult in some cases. For example, Tommasi et al. reported a case of *L. casei *bacteremia in a 66-year-old patient, which PCR only confirmed with the sequencing of 16s ribosomal RNA of the bacteria [18]. Since *L. casei* is also found in the human gut as normal flora, there remains a possibility of direct translocation from the gut into the blood that we could not eliminate.

## Conclusions

The lessons to be learned from this case include the importance of considering *Lactobacillus casei *as a potential causative agent of endocarditis in immunocompromised patients and in patients with underlying heart conditions and taking probiotics, along with the need for clinicians to be aware of the diagnostic challenges in distinguishing between a contaminant and a causative organism. The increasing utilization of probiotics demands the establishment of methods for the routine detection of microorganisms that may cause sepsis in risky populations. The presentation of the patient was also not typical of infective endocarditis. No classical signs of infective endocarditis were identified. In this case, one could never rule out endocarditis as the cause of the illness based on the symptoms and signs only. This case shows that infective endocarditis can present in various forms, which should be suspected, especially in immunocompromised patients who use probiotics. This case also highlights the importance of surgical intervention in patients with large vegetations or abscesses.
